# Phenotypical and Genotypical Comparison of *Clostridium difficile* Isolated from Clinical Samples: Homebrew DNA Fingerprinting versus Antibiotic Susceptibility Testing (AST) and Clostridial Toxin Genes

**DOI:** 10.1155/2021/7386554

**Published:** 2021-12-02

**Authors:** Javid Sisakhtpour, Fatemeh Savaheli Moghadam, Sepideh Khodaparast, Nima Khoramabadi, Ashraf Mohabati Mobarez

**Affiliations:** Department of Bacteriology, Faculty of Medical Sciences, Tarbiat Modares University, Tehran, Iran

## Abstract

**Background:**

*Clostridium* (*Clostridioides*) *difficile* is recognized as the major cause of healthcare antibiotic-associated diarrhea. We surveyed a molecular epidemiological correlation between the clinical isolates from two general hospitals in Iran through clustering toxigenic types and antibiotic susceptibility testing (AST) accuracy.

**Methods:**

Study population included 460 diarrhoeic specimens from inpatients with a history of antibiotic therapy. All samples underwent enriched anaerobic culture, confirmed by detection of *glu*D gene with PCR. Toxin status and AST were assessed by the disk diffusion method (DDM) and minimal inhibitory concentrations (MICs) of metronidazole, vancomycin, and rifampin. *C. difficile* outbreak was analyzed through conventional PCR by tracing toxin genes and Homebrew pulsed-field gel electrophoresis (PFGE) for characterizing isolates within our healthcare systems.

**Results:**

A total of 29 *C. difficile* strains were isolated by enriched anaerobic culture from the clinical samples. Among them, 22 (4.8%) toxigenic profiles yielded toxins A and B (*tcd*A, *tcd*B) and binary toxins (*cdt*A, *cdt*B). The minimum inhibitory concentration (MIC) was 18.1% and 9% for vancomycin and metronidazole, and all isolates were susceptible to rifampicin and its minimum inhibitory concentration was at <0.003 *μ*g/mL. The most dominant toxigenic and antibiotic-resistant “pulsotype F” was detected through PFGE combined with multiple Clostridial toxigenic pattern and AST.

**Conclusions:**

DNA fingerprinting studies represent a powerful tool in surveying hypervirulent *C. difficile* strains in clinical settings. Resistance to vancomycin and metronidazole, as first-line antibiotics, necessitate accomplishment of proper control strategies and also prescription of tigecycline as a more appropriate option.

## 1. Introduction

*Clostridium difficile* (new taxonomic name: *Clostridioides difficile*) is a Gram-positive, spore-forming, obligate anaerobe and recognized as the most common cause of nosocomial and gastrointestinal infections such as mild diarrhea, severe pseudomembranous colitis, and toxic megacolon. The pathogenicity of this bacterium is related to the toxin production of A and/or B and binary toxins which are encoded by *tcd*A, *tcd*B, and *cdt*A and *cdt*B genes [1, 2].

*C. difficile* infection (CDI) is initiated following antimicrobial consumption and eventuates in disruption of the normal colon microflora. Antibiotic therapy may also cause *C. difficile* antibiotic resistance in patients suffering from CDI and is a source of high morbidity and mortality worldwide.

Epidemiological studies of *C. difficile* in European countries have shown an increase in infection rate over time. CDI incidence in the United States in 2011 was more than 400,000 cases and resulted in 29,000 deaths in patients mostly aged above 65 years. CDI mortality rate before the year 2000 was low with a rate of less than 2%; however, it has since increased to 16.7% [3, 4].

The severity of CDI in the world is influenced by overuse of antibiotics (especially fluoroquinolones, macrolides, and *β*-lactams), prolongation of hospitalization, and increase in the aging population [5, 6].

The easy transmission of *C. difficile* through the oral-fecal route results in high persistence in the environment and is a major hospital problem. Early detection of CDI is important to prevent the transmission of the organism in clinical settings, as well as in managing the prescription of antibiotics [7].

Although the first-line treatments for CDI are metronidazole and vancomycin, fidaxomicin is also considered a complementary therapy. However, several cases of resistance to metronidazole and vancomycin have been reported around the world [8, 9]. Determination of antimicrobial resistance patterns is critical for both patient treatment and epidemiological studies.

For instance, reports of outbreaks from Canada, the United States, Asia, and the UK confirmed that fluoroquinolone resistance was related to the emergence of *C. difficile* NAP1/027/BI [5, 10].

According to studies from Iran by Goudarzi et al., the resistance to metronidazole and vancomycin was 5.3% and 8%, respectively [11]. However, Shoaei et al. reported 100% of isolates to be susceptible to metronidazole and 11.7% of isolates to be resistant to rifampicin in 2019 [10]. Unlike the several epidemiological investigations reported from Europe, North America, and Australia, there are limited studies in Middle East Asia [12].

Metronidazole (nonsevere CDI) and vancomycin (severe CDI) are the first-line treatments of CDI. Although vancomycin was more effective than metronidazole for chemotherapy of severe and mild/moderate CDI, several cases of resistance to metronidazole and vancomycin have been reported from around the world [8, 9, 13].

Some antibiotics that are mostly related to *C. difficile*-associated disease (CDAD) are clindamycin, ampicillin, and cephalosporin, which may contribute as important risk factors for the progress of CDAD [12, 14, 15]. Therefore, the emergence of metronidazole-nonsusceptible *C. difficile* is a serious concern in clinical settings [16].

Furthermore, multidrug-resistant (MDR) strains have emerged owing to the uncontrolled usage of antibiotics. Hence, disclosure of susceptibility profile is a strategy toward lowering the increasing antibiotic resistance trend.

In the present study, molecular epidemiology of *C. difficile* infection in the two general hospitals of Tehran was characterized by pulsed-field gel electrophoresis (PFGE), possession of A and B toxin and binary toxin genes, and also antimicrobial susceptibility pattern.

## 2. Materials and Methods

### 2.1. Study Population

A total of 460 clinical stool samples were collected during the years 2017 to 2019. Diarrhoeic stool samples belonged to adult patients with a history of antibiotic therapy from 2 to 8 weeks, followed by symptomatic antibiotic-associated diarrhea [17].

**Figure 1:**
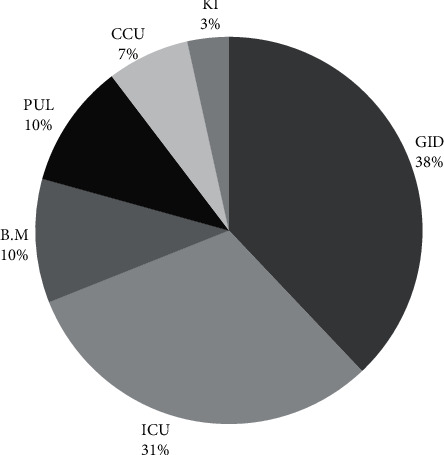
Prevalence of *Clostridium difficile* infection between inpatients of critical hospital wards (GID: gastroenterology, ICU: intensive care unit, BM.: bone marrow transplantation, PUL: pulmonary disease, CCU: cardiac surgery, and KI: renal disorder).

The study population was hospitalized in several wards, e.g., the intensive care unit (ICU), bone marrow transplantation, gastroenterology, cardiac surgery, renal disorder, and pulmonary disease (Figure 1).

### 2.2. Enriched Toxigenic Culture and Identification *C. difficile*

The fecal specimens were transported at room temperature and cultured anaerobically within 8 hr of collection or stored at 4°C for no more than 48 hr. Toxigenic culture was performed after isolation of *C. difficile* [18]. One portion of each sample was cultured regularly, and the rest was exposed to alcoholic shock for 1 hr before being cultured to inhibit the growth of other bacteria in feces [19].

Treated and untreated samples were inoculated onto the cycloserine-cefoxitin fructose agar, enriched with vitamin K_1_ solution (1 mg/mL and hemin solution 5 mg/mL), placed in anaerobic jars (Merck) with a Gas Pack Anaerocult® A (Merck, Germany), and incubated at 37°C for 2–5 days [20].

To optimize the growth of *C. difficile*, suspicious colonies were subcultured under anaerobic condition into BHI agar supplemented with 5% (v/v) sheep blood and incubated at 37°C for 24 hr. BHI agar was used for investigating colony characteristics (flat, horse barn odor, and Gram staining) and DNA extraction. Molecular identification of *C. difficile* was performed by conventional PCR of specific gene glutamate dehydrogenase (*glu*D). Confirmed colonies were preserved at −80°C for long-term storage.

### 2.3. DNA Extraction

Preserved *C. difficile* isolates were transferred with an inoculating loop into a 1.5 mL microcentrifuge tube containing 200 mL of sterile PBS buffer. Total bacterial DNA was extracted by using the QIAamp kit (Qiagen, Germany), according to the manufacturer's instructions.

### 2.4. Detection of *C. difficile* Toxin Genes

To detect enterotoxin (*tcd*A) and cytotoxin (*tcd*B) and binary toxin (*cdt*A*, cdt*B) genes, endpoint PCR was performed on DNA extracted from *C. difficile* isolates. The primers are shown in Table 1. PCR amplification was conducted as described in a previous study. In brief, thermocycler condition covered denaturation at 94°C for 10 min, 30 cycles of 94°C for 50 s, 52°C for 50 s, and 72°C for 50 s, with a final extension at 73°C for 10 min [21–23].

### 2.5. Antibiotic Disks

Disk diffusion was performed with the following material: antibiogram disks obtained from Merk, Germany, and ROSCO, Denmark. Minimum inhibitory concentration (MIC) test strips were bought from Liofilechem, Italy.

### 2.6. Antibiotic Susceptibility Test

Antibiotic susceptibility to vancomycin (VAN), metronidazole (MTZ), rifampicin (RA), tigecycline (TIG), ciprofloxacin (CP), erythromycin (E), clindamycin (CC), amoxicillin-clavulanate (AMC), tetracycline (TET), meropenem (MEN), imipenem (IMI), and chloramphenicol (C) was determined using the disk diffusion method as per clinical laboratory standards EUCAST breakpoints 2021. Results classified isolates as susceptible, intermediate, and resistant strains.

MIC of vancomycin was determined by the agar dilution method and MIC for metronidazole was determined by the test strips (Liofilechem, Italy) method as recommended by the EUCAST breakpoints on Brucella blood agar supplemented with 5% sterile sheep blood, 5 *μ*l/mL hemin, and 1 *μ*l/mL vitamin K_1_, after 24 hr of incubation at 37°C in the anaerobic jar (Gas Pack Anaerocult® A Merk, Germany) [24].

MIC values were tested using the following MIC ranges: vancomycin >2 mg/L and metronidazole >2 mg/L, based on the EUCAST breakpoint [25]. An agar plate without any antimicrobial agent was permanently incubated as growth control. An isolated *C. difficile* colony was tested for susceptibility to vancomycin and metronidazole by the agar dilution method [26]. Double dilution of each antibiotic was conducted in 1280 *μ*g/mL of stock solution, and it was added to enriched Brucella agar with 5 *μ*l/mL hemin, 1 *μ*l/mL vitamin K_1_, and 5% (v/v) sheep blood.

Plates with double concentration of antibiotics were prepared, namely, 0.25–16 *μ*g/mL for vancomycin, 0.0002–32 *μ*g/mL for rifampicin, and 0.12–64 *μ*g/mL for metronidazole. The suspension equivalent of *C. difficile* 0.5 McFarland standard was prepared in Brucella broth. The MIC results were read after 48 hr of incubation at 37°C under anaerobic condition (Gas Pack Anaerocult® A Merk, Germany).

Antibiotic susceptibility was defined as vancomycin breakpoint, >2 *μ*g/mL based on the EUCAST guideline, >2 *μ*g/mL for metronidazole breakpoint based on EUCAST breakpoints [27], and 0.004 *μ*g/mL for rifampicin as described previously (tested for epidemiological purpose only) [28].

### 2.7. DNA Fingerprinting

Fingerprinting of DNA was performed by the pulsed-field gel electrophoresis (PFGE) technique to discriminate between strains. Firstly, bacteria were cultured in BHI agar with 5% (v/v) sheep blood under anaerobic condition, overnight at 37°C. A suspension of bacteria was made in cell suspension buffer (100 mM Tris, 100 mM EDTA, pH 8.0) with the absorbance range (optical density) of 1.4–1.7. The fresh bacterial cells were harvested from the 5 mL broth culture by centrifugation for 5 min at 8000*g*. Then, 300 *μ*L of Gram-positive lysis buffer (6 mM Tris, 1 M NaCl, 100 mM EDTA, 0.5% Brij-58, 0.2% sodium deoxycholate, and 0.5% sodium lauryl sarcosine) was added to bacterial cell suspension and gently mixed. The suspension contained bacteria cells and Gram-positive lysis buffer mixed in a 1 : 1 ratio to molten 1.8% PFGE-grade, low-melting-point agarose to make plugs. The plug was then put into 3 mL of Gram-positive lysis buffer with RNase (1 mg/mL) and lysozyme (5 mg/mL) and incubated overnight at 35°C. The following morning, the buffer was replaced with a fresh solution containing proteinase K, EDTA 0.5 M, and sodium dodecyl sulfate incubated overnight in a 55°C shaking bathwater. Next day, the plugs were washed in TE buffer (Tris 1M, EDTA 0.5 M) and digested with *Sma*I restriction enzyme (20 U/plug) by overnight incubation at 25°C. The digested plug was run in a 1% PFGE-grade agarose gel using the CHEF Mapper system (Bio-Rad Laboratories, Inc.) with the following settings: Int.Sw.Tm = 5 s, Fin.Sw.Tm = 40 s, run time = 18 h, gradient = 6 and included angle = 120 [29]. The gels were analyzed with Bionumerics software (Applied Maths, GelCompar II, Belgium) to develop a dendrogram.

## 3. Results

### 3.1. Clinical Data of Patients

During this study, we screened 460 patients who were suspicious of CDI. Twenty-nine (6.3%) stool samples were positive in culture and confirmed as *C. difficile* through endpoint PCR for *glu*D. Intended patients were hospitalized in the gastroenterology (*n* = 11), intensive care unit (ICU) (*n* = 9), bone marrow transplantation (*n* = 3), pulmonary disease (*n* = 3), cardiac surgery (*n* = 2), and renal disorder (*n* = 1) wards (Figure 1).

Of 29 positive cultures, 16 patients were female (55.1%) and 13 were male (44.8%) and the average age was 54.3 years. Analysis of patient history demonstrated that the medium hospitalization duration was 17.1 days and 60% of patients used at least 3 antibiotics before sampling. The frequent antibiotics administered were meropenem (79.3%) and vancomycin (48.2%) (Table 2).

### 3.2. Toxigenic Profile

Detection of toxin A (*tcd*A) and toxin B (*tcd*B) genes was performed by conventional PCR. In total, 29 (6.3%) *C. difficile* isolates were yielded through anaerobic culture, in which 22 (4.8%) were toxigenic, 20 isolates were (*tcd*A+, *tcd*B+), 2 (0.4%) isolates were (*tcd*A−, *tcd*B+), and also 7 (1.5%) isolates were nontoxigenic (*tcd*A−, *tcd*B−). Furthermore, 3 (0.7%) isolates possessed binary toxin genes (*cdt*A and *cdt*B) (Table 3).

### 3.3. Antibiotic Susceptibility Tests

We used the agar dilution method to assess the minimum inhibitory concentration of vancomycin, metronidazole, and rifampicin in toxigenic isolates. The MIC50 and MIC90 results for the three antibiotics are demonstrated in Table 4. Metronidazole and vancomycin resistance was shown in 9% and 18.1% of isolates, respectively, while all isolates were susceptible to rifampicin and the minimum inhibitory concentration was at <0.003 *μ*g/mL.

In addition, the disk diffusion method for AST showed that most isolates were susceptible to tigecycline (100%), metronidazole (83.3%), vancomycin (77.7%), and rifampicin (75%). The susceptibility rates for other antibiotics included chloramphenicol (88.8%), tetracycline (52.7%), amoxicillin-clavulanate (38.8%), imipenem (25%), meropenem (21.1%), clindamycin (13.8%), ciprofloxacin (13.8%), and erythromycin (8.3%).

### 3.4. Multidrug Resistance (MDR)

MDR indicates resistance to one agent in three or more antibiotic classes. High-level resistance to ciprofloxacin was detected in most of the *C. difficile* isolates. Two (5.5%) isolates were MDR and exhibited resistance to vancomycin, metronidazole, and ciprofloxacin.

### 3.5. PFGE

**Figure 2:**
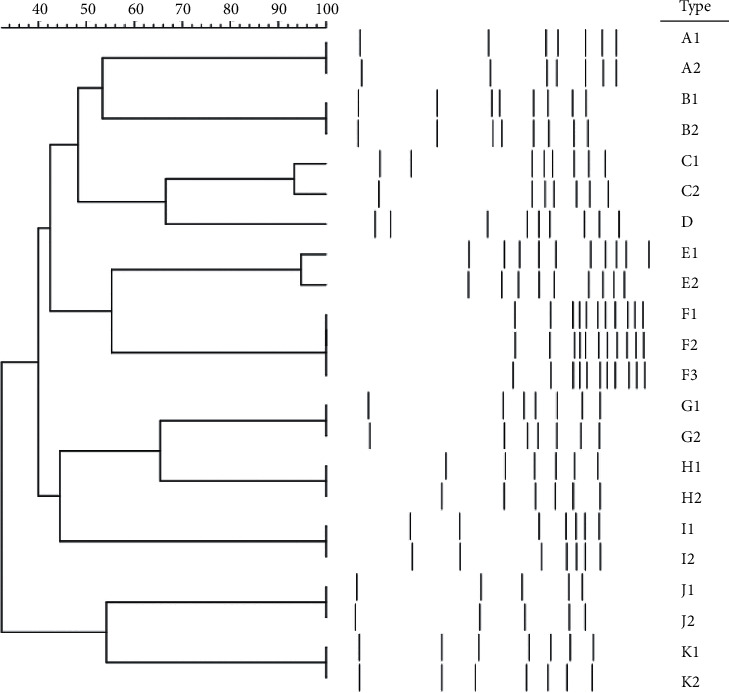
Dendrogram showing PFGE types and their correlations between isolated toxigenic or nontoxigenic *Clostridium difficile* strains.

A dendrogram, produced from PFGE data, demonstrated 22 toxigenic isolates divided into 11 clusters and 13 subtypes (based on a similarity value of 0.80) (Figure 2). The most dominant type was pulsotype F which was identified in 3 (13.6%) isolates from gastroenterology and ICU wards. Pulsotype F was toxigenic with (*tcd*A+/*tcd*B+) and binary toxin (*cdt*A and *cdt*B) genes. Twenty (*tcd*A+/*tcd*B+) isolates had 10 different pulsotypes and 12 subtypes. These pulsotypes were identified in gastroenterology and ICU wards in both hospitals. All of the isolates that distinguished into 4 pulsotypes were screened in the ICU ward. Two (*tcd*A−/*tcd*B+) isolates showed the same pulsotype which belonged to the ICU ward. Both types C1 and K2 showed concurrent resistance to metronidazole and vancomycin; these types were isolated from gastroenterology and ICU wards.

### 3.6. Statistical Analysis

The results were analyzed through one-way analysis of variance (ANOVA) and pairwise two-tailed correlation with SPSS Version 25.0 (IBM® SPSS® Statistics, USA).

## 4. Discussion

In the last decade, with increasing nosocomial diarrhea among people in North America and Europe, CDI has become a major problem [30]. However, the epidemiology of CDI is less known in Asia in general and the Middle East, in particular [31]. In this study, 460 suspicious patients were evaluated for *C. difficile* infection, antibiotic resistance pattern, and molecular characteristics. PFGE was performed to demonstrate the epidemiological characteristics of *C. difficile* isolates in our local health systems.

CDI prevalence in our study was 4.8% (22/460), comparable to the studies from the United States and Europe [4]. The prevalence of CDI in Kuwait and Qatar was reported to be 7.2% and 7.9%, respectively [32, 33]. In a survey performed in Saudi Arabia, the incidence of CDI was 1.7 per 10,000 patients [34]. The annual CDI prevalence in Iran in the years 2017 and 2019 was 18.1% and 11.4%, respectively [10, 33].

The risk factors associated with CDI include old age (≥65 years), antibiotic consumption, hospitalization, and exposure to healthcare systems [35]. Our sample population had been exposed to antibiotics for 2 months prior to the study (Table 2), and the mean duration of hospitalization was 17.1 days. Analysis of the patients' history demonstrated that beta-lactams were the most common antibiotics before the occurrence of CDI. Our study reported antibiotics therapy panels including beta-lactams, fluoroquinolones, and lincosamides.

Although metronidazole and vancomycin are the current choices for treatment of mild-to-moderate CDI and severe infection, susceptibility to these antibiotics has been gradually decreasing [13, 36, 37]. In a study conducted in Israel, the susceptibility to metronidazole and vancomycin among ribotype 027 was 44.6% and 87.7%, respectively [38]. A study of antimicrobial resistance among toxigenic *C. difficile* isolates in Iran in 2013 showed resistance to metronidazole and vancomycin to be 5.3% and 8%, respectively [11]. Also, current studies in Iran showed a susceptibility decrease to all antibiotics [10].

In our study, the resistant phenotype was observed in 5.5% isolates. The MIC90 for metronidazole was 1 mg/L. However, 77.2% of isolates were inhibited in <1 mg/L concentration of metronidazole and 2 isolates were resistant to >256 mg/L of metronidazole. According to data from the present study, up to 81% of isolates were inhibited with 1 mg/L of vancomycin. However, 4 isolates were resistant to vancomycin (MIC was 4 mg/L for two isolates and 8 mg/L for two isolates).

The percentage of MDR *C. difficile* ranges from 2.5% to 66% in various countries. Noticeably, resistance to vancomycin and metronidazole is a great concern that necessitates a proper consumption route.

Previous studies from Iran's neighboring countries report low resistance to metronidazole and vancomycin as assessed by disk diffusion assay and MIC. In addition, in East Asian and European countries, the rate of resistance to these antibiotics has been low (0–6.3%) as confirmed by various methods. Owing to the high-level metronidazole resistance, its prescription and consumption should be confined.

Based on disk diffusion assays, all isolates were susceptible to tigecycline. The majority of isolates were susceptible to commonly prescribed agents based on both the antibiotic susceptibility test and MIC results.

In the present study, the MIC50 for vancomycin was 1 mg/L and MIC90 was 8 mg/L, breakpoint to vancomycin was MIC >2 mg/L, and 4 isolates were vancomycin-resistant. The MIC50 and MIC90 of metronidazole were 0.5 mg/L that was significantly lower than the susceptibility category breakpoint of ≥32 mg/L. Only two isolates were resistant to metronidazole with MIC ≥265 mg/L.

Toxigenic and drug-resistant *C. difficile* has been reported in various regions of the world. Accordingly, an urgent antibiotic susceptibility test report is essential alongside pathogenicity assessment to avoid the selection of nonsusceptible isolates.

Therefore, based on previous research studies on susceptibility to metronidazole and vancomycin, a subinhibitory concentration of these antibiotics can promote the production of biofilms and the resistance to metronidazole and vancomycin in *C. difficile* isolates. In case of failure of antibiotic therapy, tigecycline has been proved highly effective [39, 40].

Furthermore, resistance to metronidazole and vancomycin may be due to overuse of these antibiotics in patients. According to our study, 16.6% and 15.5% of patients had a history of usage metronidazole and vancomycin, respectively, and generally, 60% of the patients used at least three prior antibiotic therapies.

Our dendrogram analysis showed that PFGE type F was the most common pulsotype identified (13.6%). All the patients harbouring pulsotype F were positive for binary toxins (*cdt*A and *cdt*B) and also *tcd*A and *tcd*B genes with a high genetic correlation. These patients were hospitalized in two different wards in the same hospital.

In the present study, genetic diversity among 22 toxigenic *C. difficile* strains was high and isolates had a low genetic correlation with each other. In addition, both pulsotypes C and K (4 isolates) were vancomycin-resistant types, but they had a low genetic correlation. Isolates in pulsotypes C and K were detected in different wards in a hospital, namely, gastroenterology, ICU, and BMT wards. A−B+ toxigenic genotype was observed in 2 isolates, belonging to pulsotype A, and these were obtained from the ICU. This pulsotype was completely susceptible to metronidazole, vancomycin, and rifampicin. It is noteworthy that pulsotypes with A−B+ toxin gene were different in our study from that of Goudarzi et al. [11].

## 5. Conclusion

Our study of adult inpatients covered antibiogram pattern and showed low correlation genetic diversity in the *C. difficile* toxin profile. Our findings highlight the necessity for continuous monitoring of the clinical history of the inpatients and antibiotic treatment procedures. It is noteworthy that our analysis was limited by the lack of strain diversity and could be improved by including more hospitals. Furthermore, the assumption of clonal transmission between present pulsotypes proved false. Finally, high susceptibility to tigecycline could prove useful for CDI treatment and must be investigated as an alternate therapy.

## Figures and Tables

**Table 1:** Primer sequences used for amplification of *tcd*A, *tcd*B, *cdt*A, *cdt*B, and *glu*D genes.

Gene	Primer	Sequence: 5 ⟶ 3′	Product size (bp)	Ref.
*tcd*A	TA1 TA2	5′-ATG ATA AGG CAA CTT CAG TGG-3′ 5′-TAA GTT CCT CCT GCT CCA TCA A-3′	624	[21]
*tcd*B	TB1 TB2	5′-GAG CTG CTT CAA TTG GAG AGA-3′ 5′-GTA ACC TAC TTT CAT AAC ACC AG-3′	412	[21]
*cdt*A	TCA1 TCA2	5′-TGA ACC TGG AAA AGG TGA TG -3′ 5′-AGG ATT ATT TAC TGG ACC ATT TG -3′	375	[22]
*cdt*B	TCB1 TCB2	5′-CTT AAT GCA AGT AAA TAC TGA G -3′ 5′-AAC GGA TCT CTT GCT TCA GTC -3′	510	[22]
GDH	*glu*D1 *glu*D2	5′-TGT CAG GAA AAG ATG TAA ATG TCT TCG AG-3′ 5′-TTA GTA CCA TCC TCT TAA TTT CAT AGC TTC-3′	1278	[21]

**Table 2:** Percentage of antibiotic classes used for treatment in 2–8 weeks prior sampling.

Antibiotics	%
*β*-Lactams	
Meropenem	27.4
Imipenem	15.5
Ceftriaxone	9.5
Cefepime	2.3
Ceftazidime	1.2
Ampicillin/sulbactam	1.2
Piperacillin	1.2
Glycopeptides	
Vancomycin	16.6
Metronidazole	
Metronidazole	15.5
Colistin	4.8
Lincosamides	
Clindamycin	3.6
Fluoroquinolone	
Ciprofloxacin	1.2

**Table 3:** Distribution of toxigenic profile of comparison isolates together with antibiotic susceptibility.

Pulsotype	Toxigenic profile genes				Antibiotic susceptibility		
	*tcd*A	*tcd*B	*cdt*A	*cdt*B	Van	Mtz	Rif
A1	+	−	−	−	S	S	S
A2	+	−	−	−	S	S	S
B1	+	+	−	−	S	S	S
B2	+	+	−	−	S	S	S
C1	+	+	−	−	**R**	S	S
C2	+	+	−	−	**R**	**R**	S
D	+	+	−	−	S	S	S
E1	+	+	−	−	S	S	S
E2	+	+	−	−	S	S	S
F1	+	+	**+**	**+**	S	S	S
F2	+	+	**+**	**+**	S	S	S
F3	+	+	**+**	**+**	S	S	S
G1	+	+	−	−	S	S	S
G2	+	+	−	−	S	S	S
H1	+	+	−	−	S	S	S
H2	+	+	−	−	S	S	S
I1	+	+	−	−	S	S	S
I2	+	+	−	−	S	S	S
J1	+	+	−	−	S	S	S
J2	+	+	−	−	S	S	S
K1	+	+	−	−	**R**	S	S
K2	+	+	−	−	**R**	**R**	S

Van: vancomycin, Mtz: metronidazole, and Rif: rifampicin.

**Table 4:** Susceptibility outcomes of isolated *Clostridium difficile* by the agar double-dilution method.

Antimicrobial agent	MIC method (*n* = 22)					Break points (mg/L)
	Antibiotics concentration range (mg/L)	S %	R %	MIC50%	MIC90%	
Vancomycin	**0.25–16**	**81.9**	**18.1**	**0.5**	**4**	**>2**
Metronidazole	**0.12–64**	**90.9**	**9.1**	**0.5**	**1**	**>2**
Rifampicin	**0.0019–32**	**100**	**0**	**0.0019**	**0.003**	**≥4**

## Data Availability

Further information is available from the corresponding author upon request (Ashraf Mohabati Mobarez (Ph.D.) Professor of Bacteriology, Department of Bacteriology, Faculty of Medical Sciences, Tarbiat Modares University Al-e Ahmad Exp. Tehran, Iran; PO box: 14115-111; mmmobarez@modares.ac.ir; office tel/fax: +98 21 8288 3862).
